# Progesterone induces neuroprotection following reperfusion-promoted mitochondrial dysfunction after focal cerebral ischemia in rats

**DOI:** 10.1242/dmm.025692

**Published:** 2017-06-01

**Authors:** Syed Suhail Andrabi, Suhel Parvez, Heena Tabassum

**Affiliations:** 1 Department of Medical Elementology and Toxicology, Jamia Hamdard (Hamdard University), New Delhi 110062, India; 2 Department of Biochemistry, Jamia Hamdard (Hamdard University), New Delhi 110062, India

**Keywords:** Progesterone, Cerebral ischemia, Neurobehavior, Mitochondria, Apoptosis, Neuroprotection

## Abstract

Organelle damage and increases in mitochondrial permeabilization are key events in the development of cerebral ischemic tissue injury because they cause both modifications in ATP turnover and cellular apoptosis/necrosis. Early restoration of blood flow and improvement of mitochondrial function might reverse the situation and help in recovery following an onset of stroke. Mitochondria and related bioenergetic processes can be effectively used as pharmacological targets. Progesterone (P4), one of the promising neurosteroids, has been found to be neuroprotective in various models of neurological diseases, through a number of mechanisms. This influenced us to investigate the possible role of P4 in the mitochondria-mediated neuroprotective mechanism in an ischemic stroke model of rat. In this study, we have shown the positive effect of P4 administration on behavioral deficits and mitochondrial health in an ischemic stroke injury model of transient middle cerebral artery occlusion (tMCAO). After induction of tMCAO, the rats received an initial intraperitoneal injection of P4 (8 mg/kg body weight) or vehicle at 1 h post-occlusion followed by subcutaneous injections at 6, 12 and 18 h. Behavioral assessment for functional deficits included grip strength, motor coordination and gait analysis. Findings revealed a significant improvement with P4 treatment in tMCAO animals. Staining of isolated brain slices from P4-treated rats with 2,3,5-triphenyltetrazolium chloride (TTC) showed a reduction in the infarct area in comparison to the vehicle group, indicating the presence of an increased number of viable mitochondria. P4 treatment was also able to attenuate mitochondrial reactive oxygen species (ROS) production, as well as block the mitochondrial permeability transition pore (mPTP), in the tMCAO injury model. In addition, it was also able to ameliorate the altered mitochondrial membrane potential and respiration ratio in the ischemic animals, thereby suggesting that P4 has a positive effect on mitochondrial bioenergetics. In conclusion, these results demonstrate that P4 treatment is beneficial in preserving the mitochondrial functions that are altered in cerebral ischemic injury and thus can help in defining better therapies.

## Abstract (precis)

**Summary:** Progesterone treatment is beneficial in preserving the altered mitochondrial functions in cerebral ischemic injury and thus can help in defining better therapies.

## INTRODUCTION

Stroke is the leading global cause of death and disability as per reports by the World Heart Association (Avasarala et al., 2015). Ischemic stroke is the most common form of stroke, accounting for 87% of all types of strokes (Bennett et al., 2014). In spite of its high prevalence, no effective treatment is currently available that can alter the course of the disease. Tissue plasminogen factor (‘clot buster’) is the only FDA approved drug that is currently available for ischemic stroke patients, but has a narrow therapeutic time window (Peplow, 2015). Thus, there is a dire need for finding a new therapeutic strategy for stroke injury that has a prolonged treatment window.

Mitochondria play a crucial role in the pathophysiology of several neurological diseases, including stroke (Andrabi et al., 2015; Rasheed et al., 2017). Mitochondria are very susceptible to any insult, due to their critical role in energy metabolism, the production of reactive oxygen species (ROS), and apoptotic pathways (Waseem et al., 2016). The primary event in ischemia is disruption of the electron transport chain (ETC) due to the impaired delivery of oxygen and glucose (Novgorodov et al., 2016). The mitochondrial membrane potential (MMP), generated via proton transfer across the inner mitochondrial membrane for the production of adenosine triphosphate (ATP), is derailed in cerebral ischemia (Khatri and Man, 2013). Upon reperfusion, the damage becomes severe as a consequence of excess ROS accumulation in mitochondria, leading to attenuated activity of ETC components and favoring the open state of the mitochondrial permeability transition pore (mPTP) (Javadov and Kuznetsov, 2013). These pathological changes eventually cause the release of various apoptotic factors such as cytochrome *c* and apoptosis inducing factor (AIF), culminating in the initiation of an apoptosis cascade and eventually leading to cell death (Manzanero et al., 2013). Neurotransmitter-based excitotoxicity is another mechanism associated with ischemic injury (Lai et al., 2014). This excitatory neurotransmitter leads to cytosolic Ca^2+^ overload and mitochondrial swelling (Nicholls et al., 2015). This swelling causes mitochondrial permeabilization, releasing the apoptotic factors, including cytochrome *c*, and resulting in cell death in ischemia (Liu et al., 2009).

A large set of experimental evidence strongly supports the neuroprotective role of the steroid hormone progesterone (P4) in many CNS injury models (De Nicola et al., 2013). Additionally, preclinical data focused on the neuroprotective role of P4 in ischemic stroke through multiple mechanisms is available (Wong et al., 2013). In addition, clinical trials investigating the neuroprotective role of P4 have also been carried out in traumatic brain injury patients, making it an attractive pharmacological agent for the treatment of ischemic injury (Schumacher et al., 2015; Wright et al., 2014; Xiao et al., 2008). P4 can act multi-mechanically by reducing oxidative damage, inhibiting apoptosis and regulating various signaling pathways in brain damage (Deutsch et al., 2013). However, the role of the mitochondria-mediated pathway in neuroprotection provided by P4 in ischemic stroke needs to be thoroughly explored before using it as a target-based therapy. Rodent models of cerebral ischemia are important tools in experimental stroke research. Such models have proven to be instrumental for the understanding of injury mechanisms in cerebral stroke as well as to identify potential new therapeutic options.

In the current study, we evaluated the mitochondria-mediated neuroprotective effects of P4, at clinically relevant concentrations, *in vivo* in the frontal cortex of a rodent model of cerebral ischemia established in our laboratory. We examined the effects of P4 on the extent of the infarction, the neurobehavioral outcome, and neurotransmitter levels in rats subjected to transient middle cerebral artery occlusion (tMCAO), an *in vivo* model of focal ischemia. Then, to elucidate its mitochondrial mechanism of action, we examined whether or not P4 could act by reducing Ca^2+^-induced rat brain mitochondrial swelling, an index of increased mitochondrial membrane permeability. In addition, we examined whether P4 could prevent the other mitochondrial functional changes, including loss of membrane potential, and alteration of and excess ROS production. To further prove our hypothesis, we analyzed mitochondrial bioenergetics by examining the state 3 respiratory control ratio (RCR) along with some ETC components. Finally, we examined the anti-apoptotic action of P4 by elucidating the translocation of cytochrome *c* from mitochondria to cytosol through the mPTP, and thereby authenticated our findings.

## RESULTS

### Neurobehavioral analysis

**Fig. 1.**
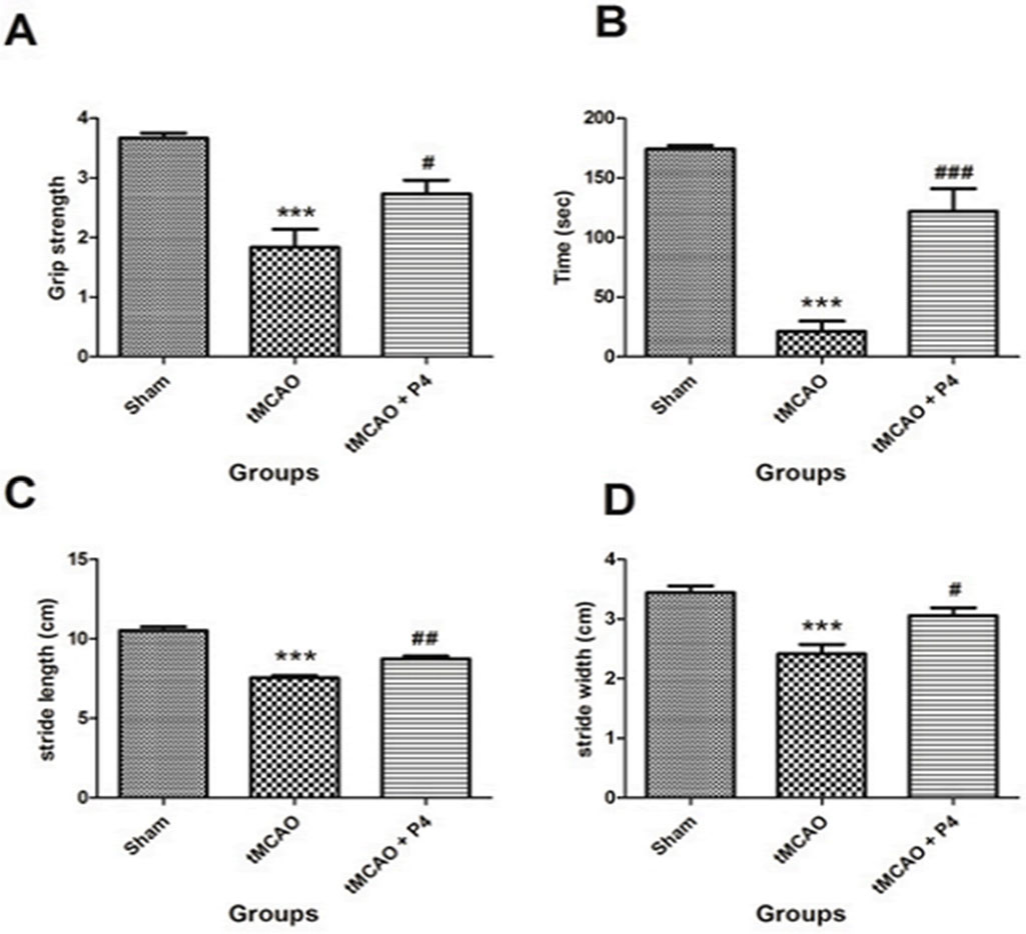
**Behavioral test parameters.** All animals were subjected to various neurological tests. (A) Grip strength test done to assess the muscular strength. ***P<0.001 versus sham, ^#^*P*<0.05 versus tMCAO. (B) Time remaining on the rotarod. ***P<0.001 versus sham, ^###^*P*<0.001 versus tMCAO. (C) Stride length to assess the gait impairment. ***P<0.001 versus sham, ^##^*P*<0.01 versus tMCAO. (D) Stride width. ***P<0.001 versus sham, ^#^*P*<0.05 versus tMCAO (*n*=6 in each group).

We studied several behavioral parameters to analyze the effect of P4 in attenuating the neurological deficits after (tMCAO) surgery. The first test involved scoring the grip strength between the sham, tMCAO and P4 administered groups. The mean reading of three successive trials for each rat was taken as a dependent variable. Grip strength decreased significantly (*P*<0.001) in rats subjected to tMCAO when compared to the sham group. Post hoc analysis showed that repeated administration of P4 [8 mg/kg body weight (b.w.)] improved the grip strength (*P*<0.05) at 24 h when compared to the tMCAO group (Fig. 1A). Next, we measured the riding time on the rotarod apparatus (in seconds) and mean of three successive trials for each rat was taken (Fig. 1B). There was a significant (*P*<0.001) performance reduction in the tMCAO group when compared to the sham group. The one-way ANOVA and post hoc analysis showed that P4 improved the rotarod performance significantly (*P*<0.001) in the treated group compared with the tMCAO group. This was followed by measurement of gait analysis of animals, which utilizes calculation of stride length and stride width. Ischemic injury induced severe gait impairment in the tMCAO group (*P*<0.001) when compared to the sham group. This was improved significantly in P4 treated rats when stride length and stride width were evaluated after treatment (*P*<0.05-0.01) when compared to the tMCAO group (Fig. 1C,D).

### P4 attenuates infarct volume after tMCAO injury

**Fig. 2.**
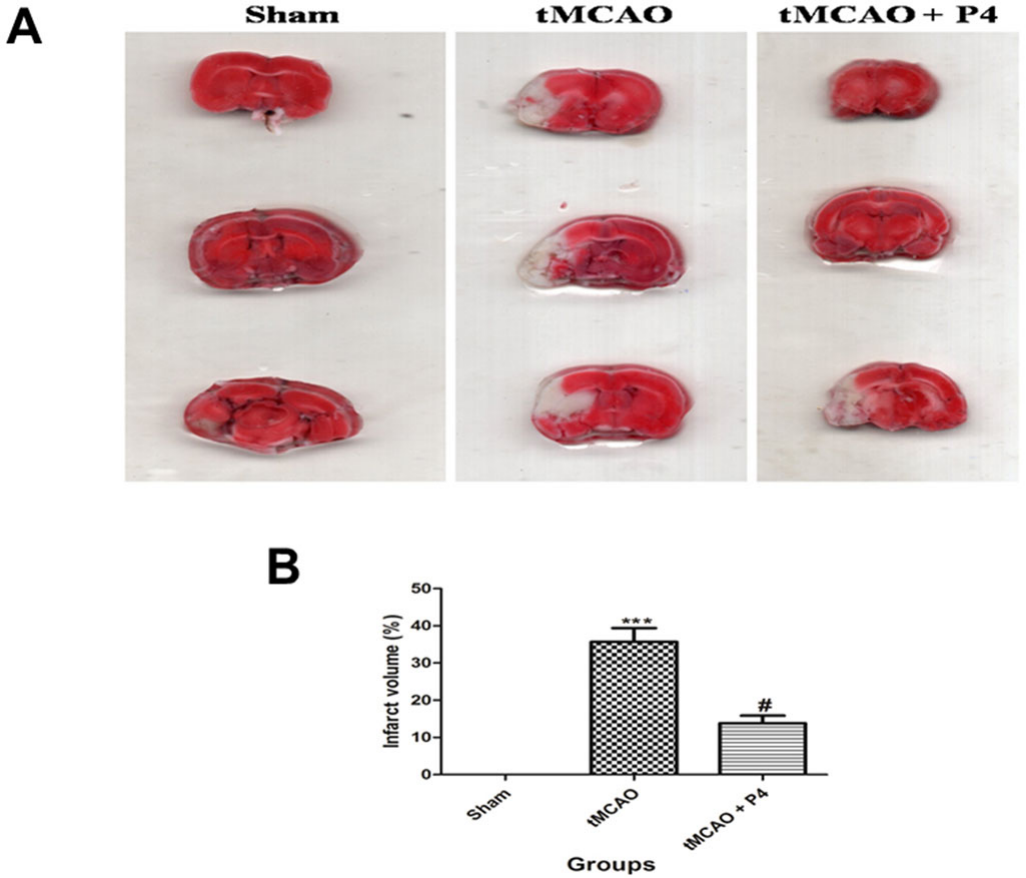
**Effect of P4 on infarct volume.** (A) Images of TTC staining. In the tMCAO group, there was severe infarction as compared to that of the P4 group. Infarcts are shown as white (unstained) regions involving cortex. (B) Infarct volume. ***P<0.001 versus sham, ^#^*P*<0.05 versus tMCAO.

In ischemic stroke models, TTC (2,3,5-triphenyltetrazolium chloride) staining is a fast and reliable visualization method for hypoxic brain tissue and dysfunctional mitochondria, and for defining the size of cerebral infarction. Brain slices from the P4-treated tMCAO group after 24 h were stained with TTC and showed a significant reduction in the infarct volume in comparison to the tMCAO rats (Fig. 2A,B).

### P4 modulates mitochondrial complexes after tMCAO

**Fig. 3.**
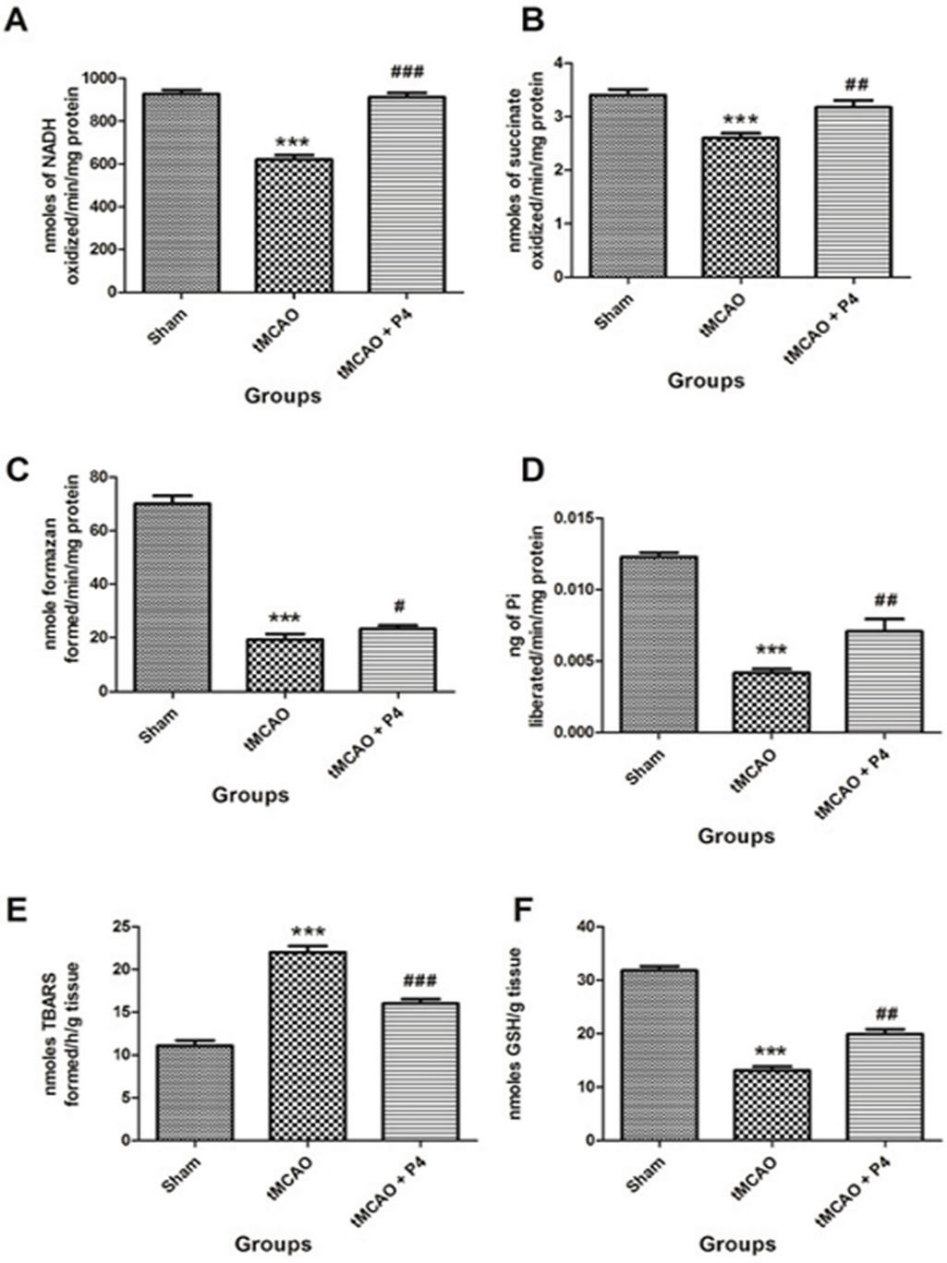
**Effect of P4 on mitochondrial complexes and oxidative parameters.** Analysis of the levels of mitochondrial complexes in isolated mitochondria from the frontal cortex of the brain (*n*=6 in each group). (A) Effect of P4 on NADH dehydrogenase (complex I). ***P<0.001 versus sham, ^###^*P*<0.001 versus tMCAO. (B) Effect of progesterone on succinate dehydrogenase (complex II). ***P<0.001 versus sham, ^##^*P*<0.01 versus tMCAO. (C) MTT assay was done to assess the metabolic activity of cells in the frontal cortex of the brain (*n*=6). ***P<0.001 versus sham, ^#^*P*<0.05 versus tMCAO. (D) Effect of P4 on F1-F0 synthase activity (complex V). ***P<0.001 versus sham, ^##^*P*<0.01 versus tMCAO. (E) Effect of P4 on mitochondrial lipid peroxidation. ***P<0.001 versus sham, ^###^*P*<0.001 versus tMCAO. (F) Effect of P4 on mitochondrial GSH. ***P<0.001 versus sham, ^##^*P*<0.01 versus tMCAO.

Mitochondrial NADH dehydrogenase (complex I) plays a crucial role in the ETC for energy production and is deregulated in ischemic-injury-induced mitochondrial dysfunction. P4 treatment significantly (*P*<0.001) elevated the activity of NADH dehydrogenase in comparison to the tMCAO-only group, where a significant (*P*<0.001) decrease in complex I was seen when compared to the sham group (Fig. 3A). A similar pattern was observed when studying another ETC complex, succinate dehydrogenase (complex II), which transports electrons to quinone and is depleted by ischemic injury. The enzyme activity of complex II was significantly (*P*<0.001) reduced in the tMCAO group when compared to the sham group (Fig. 3B). P4 administration significantly (*P*<0.01) elevated the complex II activity in the treated group when compared to the tMCAO group. Furthermore, the MTT [3-(4,5-dimethylthiazol-2-yl)-2,5-diphenyltetrazolium bromide] reduction rate (complex III) was used to assess the effect on the activity of the mitochondrial respiratory chain. P4 significantly (*P*<0.05) improved the cell viability, while a significant (*P*<0.001) decrease in cell viability in the tMCAO group was observed (Fig. 3C). Administration of P4 significantly (*P*<0.01) restored the levels of complex V, while this was significantly (*P*<0.001) decreased in the tMCAO group (Fig. 3D).

### Effect of P4 on mitochondrial oxidative stress parameters

Lipid peroxidation (LPO) is one of the important biomarkers of oxidative damage. The level of mitochondrial LPO was significantly (*P*<0.001) elevated in the tMCAO group and was significantly (*P*<0.001) reduced with P4 treatment (Fig. 3E). Glutathione (GSH) is one of the most crucial non-enzymatic antioxidants involved in rescuing cells from oxidative damage. Mitochondrial GSH levels were found to be significantly (*P*<0.001) reduced in tMCAO rats and were significantly (*P*<0.01) restored with P4 treatment (Fig. 3F).

### P4 and mitochondrial bioenergetics

**Fig. 4.**
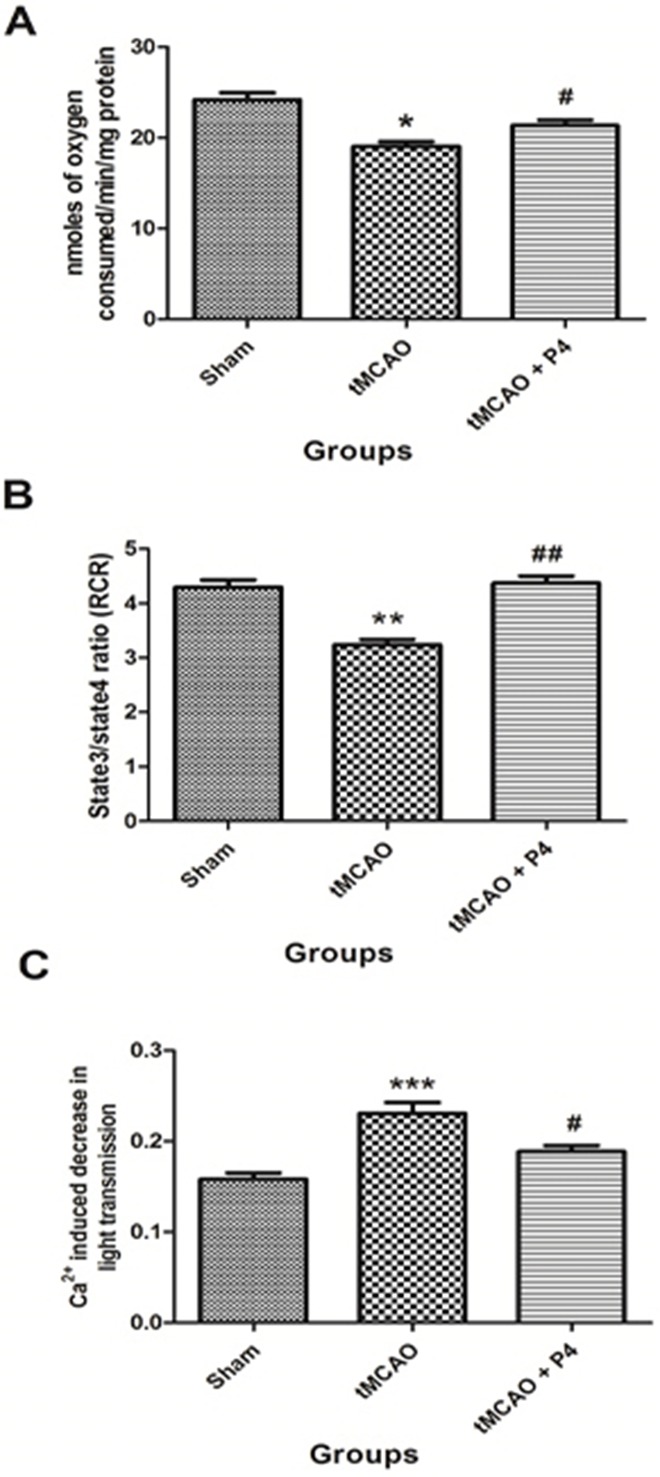
**Effect of** **P4** **on mitochondrial bioenergetics.** (A) Effect of P4 on mitochondrial oxygen consumption (state 3 respiration rate). *P<0.05 versus sham, ^#^*P*<0.05 versus tMCAO. (B) Effect of P4 on respiratory control ratio (RCR) in sham, tMCAO and tMCAO+P4 (*n*=6 in each group). **P<0.01 versus sham, ^##^*P*<0.01 versus tMCAO. (C) Effect of P4 on mitochondrial swelling. ***P<0.001 versus sham, ^#^*P*<0.05 versus tMCAO (*n*=6 in each group).

We investigated the effect of P4 on mitochondrial oxygen consumption (state 3 respiration) and the RCR at 24 h after tMCAO. Results show that oxygen consumption and RCR was reduced (*P*<0.05-0.01) in the frontal cortex of tMCAO animals when compared to the sham group. P4 at 8 mg/kg b.w. significantly (*P*<0.05-0.01) restored the oxygen consumption as well as the RCR in the tMCAO P4 treated group when compared to the tMCAO alone group (Fig. 4A,B).

### P4 attenuates mitochondrial swelling

P4 was able to bring down the levels of Ca^2+^-induced mitochondrial swelling. There was no significant difference in light transmission between any groups before adding the Ca^2+^. The baseline value was taken for 5 min after addition of Ca^2+^ at the concentration of 400 µM. A significant (*P*<0.001) decrease in light transmission in the tMCAO group occurred when compared to the sham group with addition of the Ca^2+^. This was significantly attenuated by P4 administration (*P*<0.05) when compared to the tMCAO group (Fig. 4C).

### P4 reduces the mitochondrial ROS

**Fig. 5.**
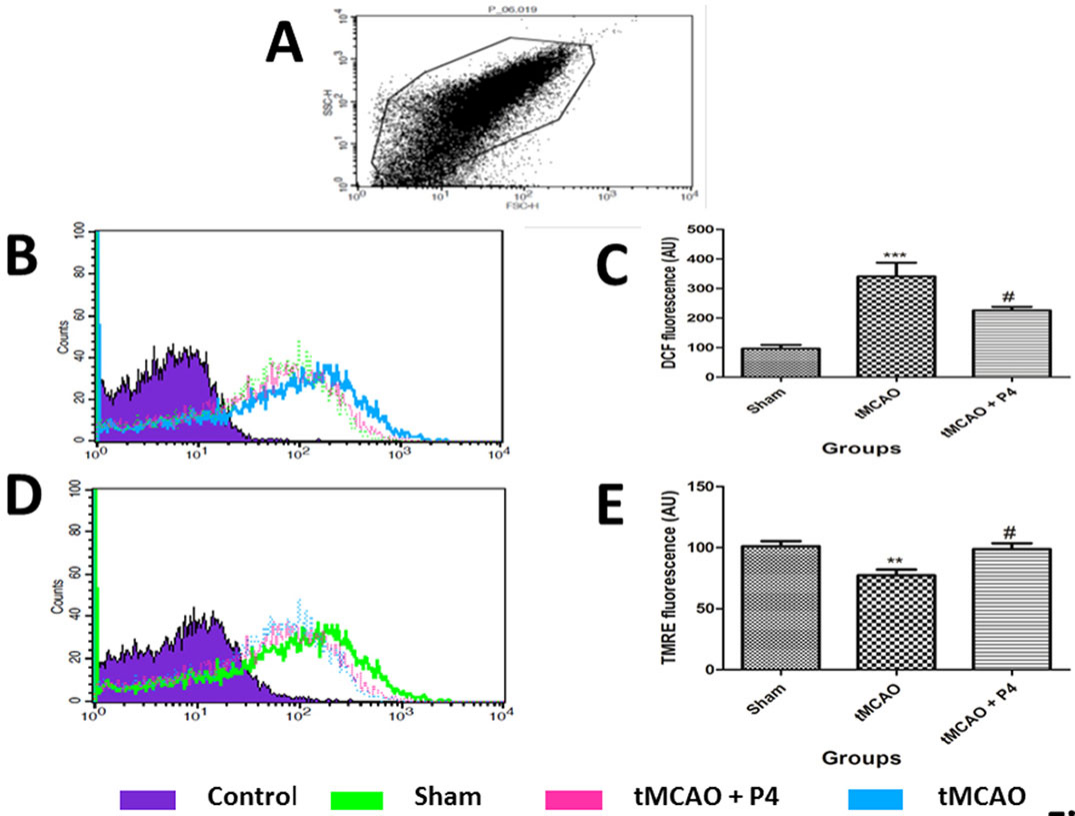
**Measurement of mitochondrial ROS and mitochondrial membrane potential (MMP).** (A) In the FSC/SSC plot of the isolated mitochondria, 10,000 events were collected within gate R1. (B) Measurement of ROS production in sham, tMCAO and tMCAO+P4 as shown by changes in DCF fluorescence. (C) The relative changes in DCF fluorescence intensity. ***P<0.001 versus sham, ^#^*P*<0.05 versus tMCAO. (D) Changes in MMP in sham, tMCAO and tMCAO+P4 as reflected by changes in TMRE fluorescence. (E) The relative changes in TMRE fluorescence intensity are shown. **P<0.001 versus sham, ^#^*P*<0.05 versus tMCAO. *n*=6 in each group.

Mitochondrial ROS production was determined by 2′,7′-dichlorofluorescein (DCF) fluorescence. Measurement of ROS production in sham, tMCAO and tMCAO+P4 mitochondria was carried out as shown by changes in DCF fluorescence intensity (Fig. 5B). There was a significant (*P*<0.001) increase in mitochondrial ROS level in the tMCAO group when compared to the sham group. P4 treatment effectively (*P*<0.05) lowered the mitochondrial ROS level as shown by reduced DCF fluorescence intensity in comparison to the tMCAO group (Fig. 5C).

### Effect of P4 on MMP

Changes in MMP in sham, tMCAO and tMCAO+P4 mitochondria was reflected by changes in TMRE fluorescence (Fig. 5D). There was a significant (*P*<0.01) reduction in MMP as indicated by low fluorescence intensity in the tMCAO group when compared to the endogenously depolarized sham group. P4 significantly (*P*<0.05) elevated the MMP as shown by high TMRE fluorescence intensity when compared to the tMCAO alone group (Fig. 5E).

### Effect of P4 on neurotransmitters

**Fig. 6.**
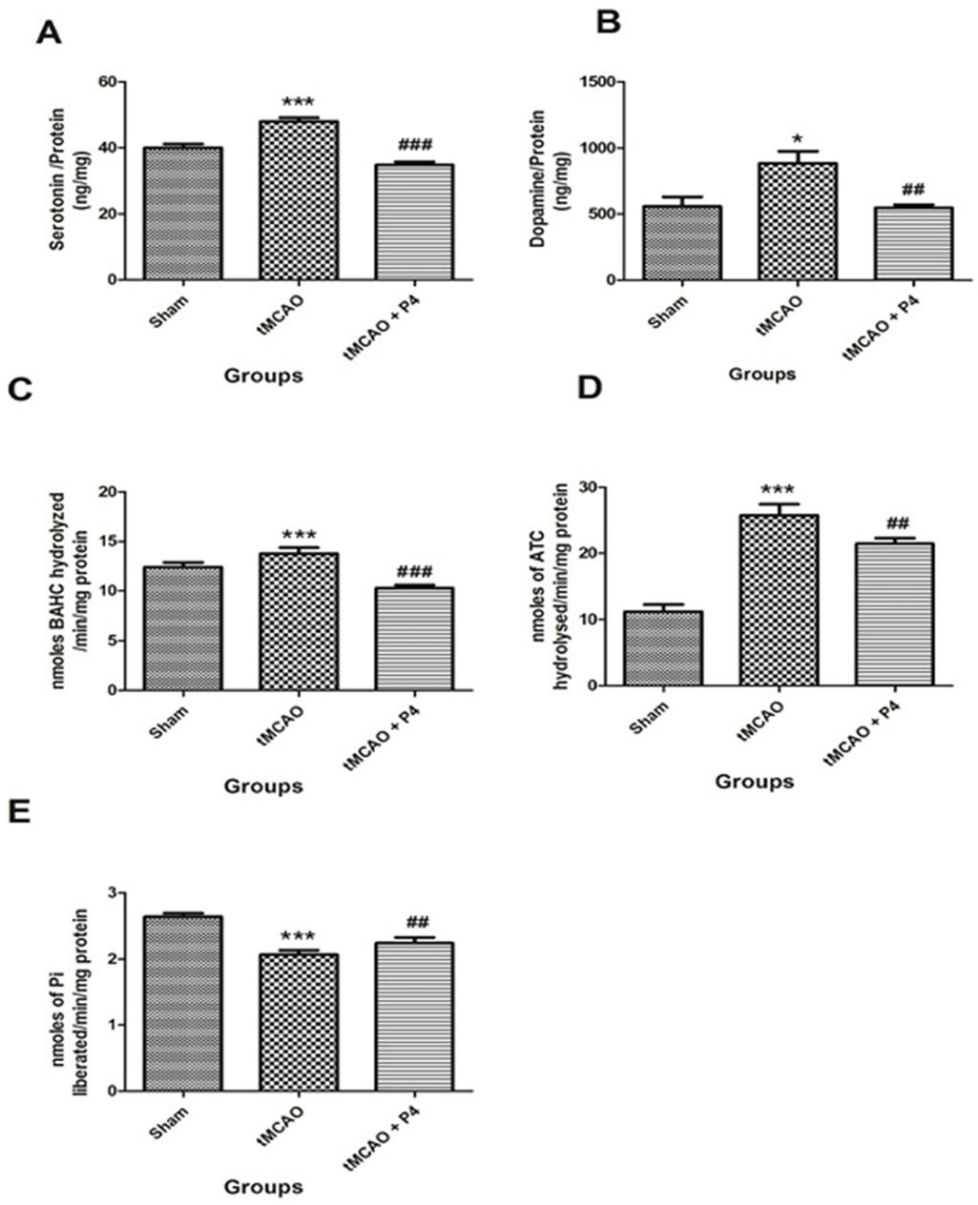
**Effect of P4 on 5-HT, dopamine and neurotoxicity parameters.** (A) Effect of P4 on 5-HT. ***P<0.001 versus sham, ^###^*P*<0.001 versus tMCAO. (B) Effect of P4 on dopamine. *P<0.05 versus sham, ^##^*P*<0.01 versus tMCAO (*n*= 6 in each group). (C) Effect of P4 on monoamine oxidase activity. ***P<0.001 versus sham, ^###^*P*<0.001 versus tMCAO. (D) Effect of P4 on acetylcholine esterase activity. ***P<0.001 versus sham, ^##^*P*<0.01 versus tMCAO. (E) Effect of P4 on Na^+^ K^+^-ATPase. ***P<0.001 versus sham, ^##^*P*<0.01 versus tMCAO (*n*=6 in each group).

P4 treatment significantly (*P*<0.001) decreased the level of serotonin (5-HT) when compared to that of the tMCAO-only group. In the tMCAO group, the level of 5-HT was observed to be significantly (*P*<0.001) raised when compared to the sham group (Fig. 6A). Also, a significant increase in dopamine level (*P*<0.05) was observed after tMCAO when compared to that of the sham group. In P4 treated animals, dopamine levels were significantly (*P*<0.01) lowered when compared to that of the tMCAO group (Fig. 6B).

### Effect of P4 on enzymatic neurotoxicity markers following tMCAO

Monoamine oxidase (MAO) is an important neurotoxicity biomarker enzyme in the brain that catalyzes the breakdown of various monoamines. P4 significantly (*P*<0.001) attenuated the level of MAO in the treatment group when compared to the tMCAO group. There was a significant (*P*<0.001) elevation in only the tMCAO group (Fig. 6C) when compared to the sham group. Another prominent neurotoxicity marker includes the enzyme acetylcholine esterase (AchE), which is crucial for synaptic termination of the nerve impulse by metabolism of acetylcholine. P4 administration significantly (*P*<0.01) lowered the activity of AchE in animals, in comparison to the tMCAO group. There was a significant (*P*<0.001) elevation of AchE enzyme level in the tMCAO group when compared to the sham group (Fig. 6D). Also, decreased levels of Na^+^ K^+^-ATPase during brain injury indicated depletion of ATP. The same was lowered significantly (*P*<0.001) in the tMCAO group, when compared to the sham group. This was observed to be restored in P4 treated animals at a significant level (*P*<0.01; Fig. 6E) when compared to the tMCAO group.

### Histopathology

**Fig. 7.**
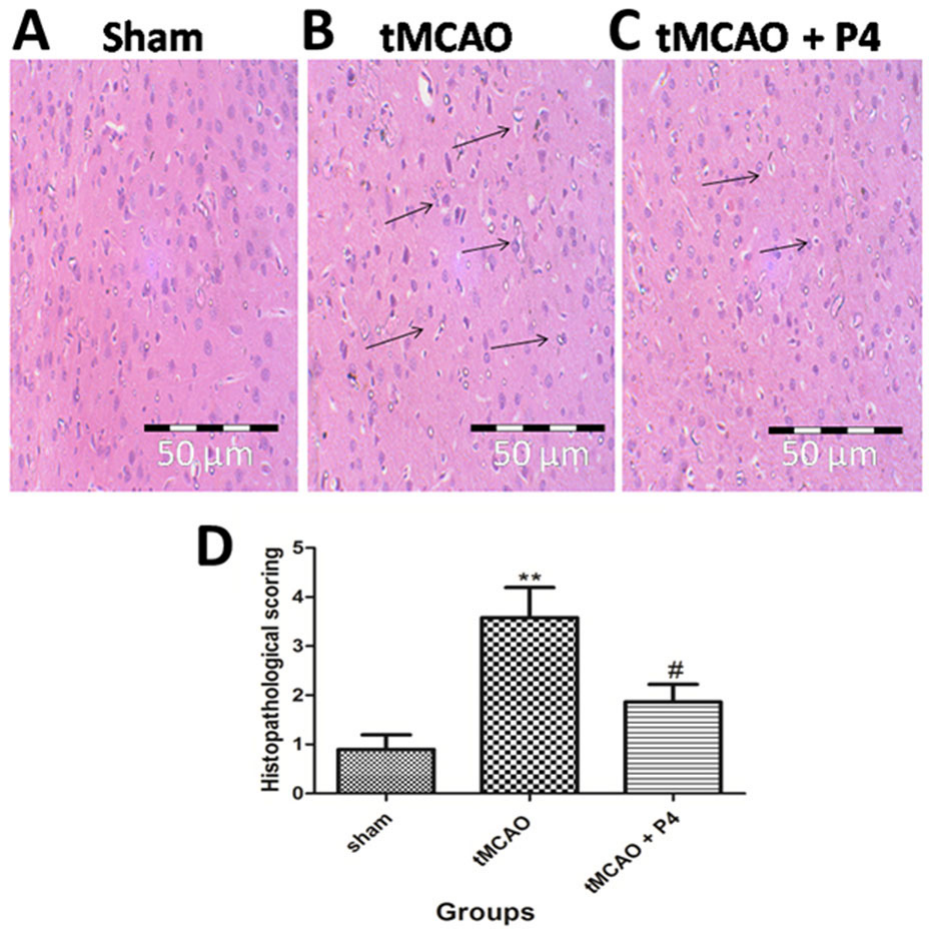
**Effect of P4 on histopathology.** (A-C) Representative histopathological photomicrograph of frontoparietal layers of the sham, tMCAO and tMCAO+P4 group. In the sham group, there was no vacuolation or any neuronal loss. In the tMCAO group, there was vacuolation and heavy neuronal loss. In the P4 treated group there was the partial neuronal loss. Magnification at 40×. (D) Histological alterations are represented graphically, showing significant changes (**P<0.001) in tMCAO and that, in the P4 treated group, there was a significant improvement (^#^*P*<0.05).

The observed histological alterations included the presence of vacuolated spaces, pyknotic nuclei and heavy neuronal loss in the tMCAO group compared to the sham group. In the P4 treated tMCAO group, there was a reduction in vacuolation and neuronal loss as compared to the tMCAO-only group (Fig. 7A-C). Fig. 7D includes graphical representation of significant histological alterations in the tMCAO group (*P*<0.001) and the P4 treated group (*P*<0.05).

### Immunohistochemical analysis of cytochrome *c* translocation

**Fig. 8.**
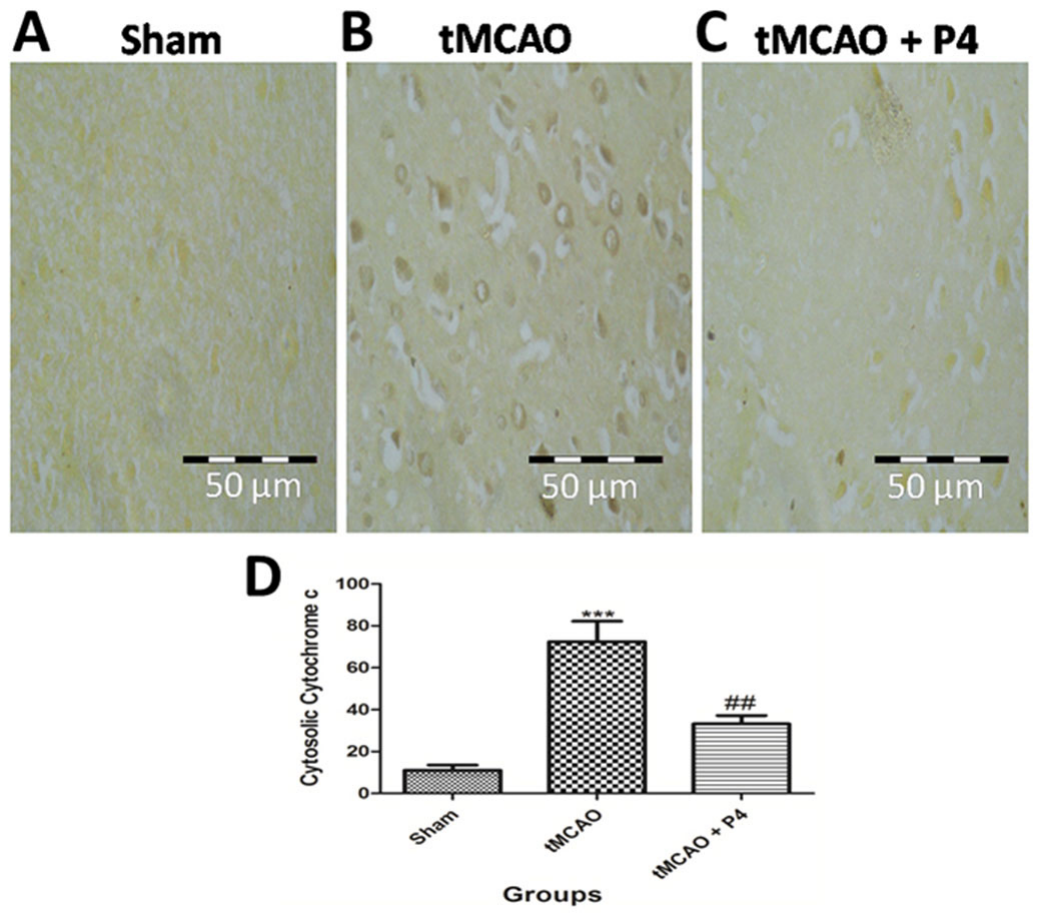
**Effect of P4 on cytochrome *c* translocation.** (A-C) Representative images of the frontoparietal layers of the brain were taken for analysis of the translocation of cytochrome *c* from mitochondria to cytosol. In the tMCAO group, the cytochrome *c* immunostaining is higher as compared to that in the sham group, which suggests cytosolic translocation of cytochrome *c*. Treatment with P4 is able to reduce the translocation of cytochrome *c* following tMCAO. (D) Quantitative measurements of cytosolic cytochrome *c* release. Cytosolic translocation of cytochrome *c* was found to be significant (***P<0.001) in tMCAO rats and its translocation was significantly inhibited (^##^*P*<0.01) with P4 treatment.

In the tMCAO group, the cytochrome *c* immunostaining was higher when compared to that in the sham group, thereby suggesting cytosolic translocation of cytochrome *c*. Treatment with P4 was able to reduce the translocation of cytochrome *c* following tMCAO (Fig. 8A-C). Cytosolic translocation of cytochrome *c* was found to be significantly (*P*<0.001) increased in tMCAO rats and its translocation was significantly inhibited (*P*<0.01) with P4 treatment. Quantitative measurements of cytosolic cytochrome *c* release are shown in Fig. 8D.

## DISCUSSION

### Importance of the study

In this study, we have investigated the potential mitochondrial mechanism underpinning the P4-enabled neuroprotection in a cerebral ischemia model with tMCAO. Based on the results obtained from evaluating alterations in various paradigms (such as mitochondrial permeability, MMP, ROS generation, and bioenergetics, including activity of ETC complexes in the frontal cortex of the brain), we can infer that mitochondrial dysfunction is one of the hallmarks of ischemia-reperfusion-induced cerebral damage leading to cellular death (Kalogeris et al., 2014). Hence, targeting mitochondria could be a promising neurotherapeutic tool (Jin et al., 2016). Blocking of the mPTP is already being considered to be one of the prime targets for protection in various neurological diseases (Fayaz et al., 2015). The main findings are: (1) P4 inhibited the formation of mPTP, eventually blocking the translocation of cytochrome *c* from mitochondria to cytosol; (2) P4 attenuated the tMCAO-induced production of mitochondrial ROS, rejuvenating the mitochondrial bioenergetics; and (3) P4 restored the activity of ETC components and various neurological functions.

### Behavioral outcomes

We have performed a number of behavioral assays in rats to support the existence of neurological deficits/anomalies associated with the cerebral ischemic condition. Muscle weakness or motor impairment is a common complaint after stroke in humans. Our results have demonstrated that tMCAO leads to severe impairment in motor coordination, which was improved with P4 treatment. Also, abnormal changes occurred in grip strength and gait patterns of tMCAO animals, and these changes were also ameliorated by repeated P4 administration at the dose of 8 mg/kg b.w. These observations are in agreement with previous findings of other research groups showing that P4 is able to improve motor coordination and various other neurological deficits (Yousuf et al., 2014). P4 treatment was also able to reduce the infarct size in animals, which can be associated with improvement of ischemia-induced neurological deficits.

### Ischemia-induced neuronal death

Infarct volume is a crucial indicator of how severe the ischemic damage is; therefore, to confirm this, TTC staining was performed, which reliably identifies the infarct core. Only tMCAO-operated animals showed significant cortically lesioned areas, thereby indicating loss of dehydrogenase activity accompanied with increased presence of ROS in the region and probable secondary excitotoxicity (Drose et al., 2016). Another supporting cause might be the inhibition of blood supply to the injured area. This was attenuated by P4 administration post-occlusion, which can be attributed to its antioxidant, anti-apoptotic, and free-radical scavenging properties (Cai et al., 2015). It can also cross the blood–brain barrier, which might have helped it in reaching the injured brain region easily (Wang et al., 2009).

### Oxidative stress causes mitochondrial dysfunction

Ischemia-induced production of ROS damages the various macromolecules and eventually may lead to cell death by apoptosis and necrosis. LPO, one of the oxidative stress markers, could lead to loss of mitochondrial membrane integrity and uncoupling of mitochondrial respiration, as was observed in our study and in a prior study by Rood and Hall (2000).

A loss of membrane integrity was observed 24 h after ischemia-reperfusion. It was accompanied by significant loss of endogenous antioxidant GSH, due to increased mitochondrial ROS as well as the compromised activity of ETC components. The change in DCF fluorescence intensity thus correlates linearly with the amount of intracellular ROS formed. Post-occlusion treatment with P4 significantly decreased the mitochondrial ROS as shown by reduction in fluorescence when compared to that of tMCAO-only rats. We assume that the antioxidant property of P4 might have helped in increasing the mitochondrial GSH content and providing protection against excess ROS (Gaignard et al., 2016). From these data, the pharmacological intervention targeting mitochondrial health seems to be a promising option for treating ischemic damage.

Due to mitochondrial LPO and oxidative stress, there was a loss of integrity of respirasomes (complexes I-V), which altered the efficacy of electron transport along the ETC (Lenaz et al., 2010). In ischemia-reperfusion, the primary event is the lack of oxygen and glucose as a result of a loss of blood supply, compromising the mitochondrial ETC complexes I-V/respirasomes (Sims and Muyderman, 2010; Sanderson et al., 2013). We made similar observations in the animals with tMCAO injury alone. P4 treatment after ischemic injury showed the elevated level of these complexes, suggesting a role of P4 in stabilizing the mitochondrial membrane.

### Modulation of mPTP: a possible neuroprotective mechanism of P4

Ischemia-reperfusion-induced mitochondrial ROS production reduces the electron transfer kinetics, which eventually reduces the MMP. MMP and synthesis of ATP can reduce in ischemic stroke due to altered respirosomes and disturbed transport of protons across the inner mitochondrial membrane (Kalogeris et al., 2012). Flow cytometric monitoring of TMRE fluorescence demonstrated that ischemic insult resulted in a quick drop in MMP, visible as a strong decline in fluorescence intensity. This was significantly improved in the P4 treated group. Moreover, mitochondrial respiration also decreased due to ischemic injury as shown by decreased state 3 respiration and RCR. P4 significantly attenuated this deficit. P4, due to its anti-oxidative property, helps in preserving respirosome assembly by protecting the mitochondrial membrane from LPO (Aggarwal et al., 2008).

Mitochondrial swelling is one of the initial post-ischemic changes (Liang et al., 2013). In the present study, we measured mitochondrial swelling as an index of mitochondrial membrane permeabilization. After an ischemic injury, there was a significant increase in mitochondrial swelling, which modulates the mPTP and enables passage of cytochrome *c* from mitochondria to the cytosol, where it forms the apoptosome. This finding led to the presumption that P4 might have blocked the mPTP by binding to cyclosporine D. However, additional molecular studies need to be done to prove this finding. We further presume that, if P4 blocks mPTP, then translocation of cytochrome *c* from mitochondria to the cytosol must have been inhibited in P4 treated rats. To prove our hypothesis, we performed immunohistochemical analysis of translocation of cytochrome *c* from mitochondria to cytosol. The translocation was lowered after P4 administration and thus this finding strengthened our hypothesis that P4 might have blocked the mPTP. This novel finding sheds light on the mitochondrial protective and anti-apoptotic role of P4 that can be utilized therapeutically in stroke injury. A previous study on isolated mitochondria has also shown that P4 and its metabolite allopregnanolone inhibit the release of cytochrome *c* from mitochondria to cytosol (Sayeed et al., 2009).

### Attenuation of neurotoxicity in ischemia by P4

We also studied the levels of brain-specific enzymes as a marker for neurotoxic damage due to ischemic injury. Alterations in the levels of these brain specific enzymes are one of the mechanisms behind neuronal cell death in ischemic stroke. Altered AchE activity and a disrupted cholinergic system play an important role in disturbed synaptic transmission and neuronal health in ischemic stroke (Kim and Kim, 2013). There was a significant elevation in AchE levels observed in tMCAO-only animals, and these were lowered with P4 administration. Studies have reported that AchE activity is increased by oxygen and nitrogen reactive species (Affonso et al., 2013). Considering that increased AchE activity has been related to progressive neurological decline and neurodegenerative diseases such as Alzheimer's and Parkinson's disease, it is presumed that a disruption in the cholinergic system may be involved in the neurological deficit associated with the tMCAO model of stroke.

We also evaluated the effect on levels of MAO, which oxidises monoamines such as dopamine, 5-HT, and adrenaline (Wąsik et al., 2014). In aging and other neurodegenerative process, an increase in the activity of the mitochondrial enzyme MAO has been seen. MAO catalyses the oxidative deamination of various biogenic and xenobiotic amines, including the monoamine neurotransmitters 5-HT and dopamine, along with generating aldehydes and H_2_O_2_ (Weinreb et al., 2016). MAO inhibitors and antidepressants are most commonly used as a treatment for post-stroke depression in stroke patients (McCann et al., 2014). Ischemia-induced injury leads to excessive release of MAO that can lead to depression and anxiety. P4 treatment was able to reduce the excess MAO levels in the tMCAO group. Another enzyme, Na^+^ K^+^-ATPase, is an ATP-dependent pump for nerve conduction, maintaining the resting potential of cell membranes (de Lores Arnaiz and Ordieres, 2014). Due to altered mitochondrial bioenergetics in ischemia, physiological processes such as the functioning of ATP-dependent ion channels can be derailed, leading to alteration of membrane potential as observed in tMCAO injury. P4 was able to substantially elevate the activity of Na^+^ K^+^-ATPase by protecting the mitochondrial damage and thus supporting the previous findings that P4 has a positive effect on mitochondrial bioenergetics.

### Changes in neurotransmitter levels affects the mitochondrial functions

This study also incorporated the analysis of neurotransmitters that play a crucial role in proper functioning of the nervous system. After the onset of ischemic stroke, cessation or reduction of blood flow to the brain induces neuronal damage. Monoamine neurotransmitters such as dopamine and 5-HT are important biogenic amines that are vital for transmitting nerve impulses for cognitive function of the body. Over-release of the above-mentioned neurotransmitters, which can be triggered by many types of neurotoxic insult, such as stroke, hazardous chemicals, tumors and neurodegenerative diseases, affect their respective receptors in the brain which can further induce cytotoxic responses, including altered calcium influx, free radical damage, oxidative stress, inflammatory responses and apoptosis, potentially leading to neuronal cell death and neurodegeneration (Chen et al., 2014).

Dopamine not only works as a neurotransmitter but acts as a good metal chelator and electron donor in *in vivo* conditions, generating toxic free radicals through the redox reaction. It has a high tendency to generate H_2_O_2_ through Fenton's chemistry. This, in turn, causes mitochondrial respiratory chain breakdown and oxidative stress leading to cardiomyopathy and neurodegeneration (Uttara et al., 2009). 5-HT has an inhibitory effect on mitochondrial respiration, causing brain ATP depletion. It causes excitotoxic death of nerve cells, which involves both limitations of energy production and increased cellular activation (Chen et al., 2014). Levels of both of these neurotransmitters were found to be elevated in the tMCAO group and were attenuated by P4 treatment. P4 is known to possess specific anti-5-HT actions (Morton et al., 2015). P4 can attenuate neuronal excitotoxicity by blocking calcium channels or by upregulating inhibitory neurotransmitter GABA, which in turn can block the release of other excitatory neurotransmitters in the CNS (Wei and Xiao, 2013). Also, we have shown in the present study that P4 inhibits the activity of the enzyme MAO, which breaks down dopamine to generate toxic products such as H_2_O_2_, oxygen-derived radicals, semiquinones and quinones, leading to mitochondrial LPO and neurotoxic effects.

### P4 improves anatomical damage in ischemic tissue

tMCAO-operated animals showed a series of histological alterations, including marked neurodegeneration with pyknotic neurons. P4 successfully ameliorated these histological alterations as observed by the reduced number of pyknotic neurons.

### Conclusion

The current study adds further evidence that P4 is neuroprotective in cerebral ischemia. Our results indicate that P4 mediates its neuroprotection through mitochondrial pathways in male rats after cerebral ischemia. In conclusion, the results demonstrate that P4 was able to modulate the functional and cellular deficits associated with ischemic injury via mitochondrial pathways. It is important to assess potential neuroprotective candidates, such as P4, in the group at highest risk of stroke. As this study focused on mitochondrial pathways in male rats, further studies are warranted to study the effects on female rats.

## MATERIALS AND METHODS

### Animals and treatment regimen

Male Wistar rats (250-300 g) were obtained from the Central Animal House Facility of Jamia Hamdard, New Delhi, India. The animals were kept under a 12 h light-dark cycle with free access to food and water. Use of animals and all experimental procedures were conducted according to the procedures approved by the Animal Ethics Committee, Jamia Hamdard.

### Experimental groups

The rats were divided randomly into three groups: (1) Sham-operated group, (2) tMCAO group, (3) tMCAO+P4 (8 mg/kg b.w.). The experimenter was blinded to the treatment group. All parameters were done in the frontal cortex of brain, and six animals were taken for each set of parameters in each group.

### Surgical procedure

Focal tMCAO was performed according to the method of Vaibhav et al. (2013). Prior to tMCAO surgery, animals were anesthetized with choral hydrate (400 mg/kg b.w.). A midline incision was made on the ventral surface of neck to expose the right common carotid artery. The external carotid artery (ECA) was ligated and internal carotid artery (ICA) was isolated near to the bifurcation. An intraluminal monofilament of filament size 4.0, length 30 mm, and diameter 0.19 mm having a silicon rubber-coated tip was introduced into the ECA and advanced through the ICA up to the origin of the middle cerebral artery (MCA). The suture was withdrawn slowly after 1 h occlusion of MCA, and rats were returned to their cages for the period of 23 h for reperfusion. In the sham group, ECA was surgically prepared but the filament was not inserted. Animals were returned to their normal environment in an air-conditioned room at an ambient temperature (25±2°C) and relative humidity (45-50%) with 12 h light/dark cycles and allowed free access to the pellet diet and purified drinking water.

### P4 treatment

P4 (P-0130; Sigma-Aldrich Co.) at a dose of 8 mg/kg b.w. was dissolved in 50% dimethyl sulfoxide (DMSO) and 50% saline, which was administered by intraperitoneal (i.p.) injection 1 h post-occlusion followed subcutaneously (s.c.) at 6, 12, and 18 h post-occlusion. This dose is considered to be the dose for producing neuroprotection (Wali et al., 2014).

### Behavioral tests

All naïve animals were given random training for 5 days for testing motor coordination, grip strength and gait pattern before the experiment in order to acclimatization and get a maximum possible score.

#### Assessment of motor impairment

To assess the sensorimotor coordination, the rats were evaluated in the rotarod task before sacrifice. In this study, motor function was assessed at 23 h post-occlusion by using a rotarod unit (Omni Rotor, Omnitech Electronics Inc., Columbus, OH, USA), which consists of a rotating rod of diameter 75 mm which is divided into four compartments to test four animals at a time (Ashafaq et al., 2012b). The time that each animal remains on the rotating rod was recorded for three trials with a minimum interval between each of 5 min and a maximum trial length of 180 s for each trial. The apparatus automatically recorded the time in tenths of a second until the rat falls to the floor. The speed was set at 10 rotations per minute and cut-off time was 180 s. The score was presented as mean of latencies of three trials on the rotating rod.

#### Assessment of grip strength

Grip test was performed by using the method of Ashafaq et al. (2012a). The apparatus consists of a string measuring about 50 cm in length, pulled tight between two vertical supports and elevated 40 cm from the flat surface. The rats were put on the string at midway and scoring was done according to the following scoring scale: 0=fall off, 1=hangs onto string by two forepaws, 2=hangs onto string by two forepaws and also tries to climb on string, 3=hangs onto string by two forepaws along with one or both hind paws, 4=hangs onto string by all forepaws along with tail wrapped around the string, 5=escape.

#### Gait analysis

The gait patterns were evaluated according to the previously published method (Ashafaq et al., 2012a) to find the gait-related anomalies at 24 h after tMCAO. Stride length and stride width were measured by using an enclosed wooden walkway of 12 cm width. The forepaws were stained with green non-poisonous coloring agent and hindpaws were stained with red color. Stride length was measured as the distance between ipsilateral forepaw and hindpaw. Stride width was taken as the side-to-side distance between the two forepaws, and the two hindpaws, respectively.

### Measurement of infarction volume

The animals were sacrificed after 1 h of occlusion followed by 23 h of reperfusion (Ashafaq et al., 2016). The brains were dissected out and kept in a brain matrix. 1.5 mm coronal sections of the brains were cut down with the help of sharp blades and stained with 0.1% TTC prepared into normal saline at 37°C for 15 min. For imaging, the sections were scanned by a high-resolution scanner. The infarct volume was measured with ImageJ software.

### Tissue collection and mitochondrial preparation

Mitochondria of brain frontal cortex of rat were isolated by the differential centrifugation method (Waseem and Parvez, 2016). Rats were decapitated and frontal cortex were dissected and homogenized by using a mechanically driven Teflon-fitted Potter-Elvehjem type homogenizer in ice-cold buffer A. Frontal cortex mitochondria were isolated in three buffers: A, B and C. Buffer A contained 250 mM sucrose, 10 mM 4-(2-hydroxyethyl)-1-piperazineethanesulfonic acid (HEPES), 1 mM EGTA, and 0.1% fat-free BSA adjusted by Tris to pH 7.4 and centrifuged at 1000 ***g*** for 8 min at 4°C. The supernatant was collected and centrifuged at 10,000 ***g*** for 10 min at 4°C. Thereafter, the obtained pellet was resuspended and washed twice with washing medium (B), containing 250 mM sucrose, 10 mM HEPES, and 0.1 mM EGTA adjusted by Tris to pH 7.4 and centrifuged at 12,300 ***g*** for 10 min. Finally, the pellet was resuspended again in an isolation medium (C), containing 250 mM sucrose, 10 mM HEPES, and 0.1% fat-free BSA adjusted by Tris to pH 7.4 and centrifuged at 12,300 ***g*** for 10 min.

### Mitochondrial complexes measurement

#### NADH dehydrogenase activity (complex I)

NADH dehydrogenase activity was assayed by spectrophotometrically according to Waseem and Parvez (2016). The enzyme activity was expressed as micromoles of NADH oxidized per minute per milligram protein using a molar extinction coefficient of 21,000 M^−1^ cm^−1^.

#### Succinate dehydrogenase activity (complex II)

The activity of succinate dehydrogenase was assayed according to the method described by Waseem and Parvez (2016) by using the spectrophotometer. The enzyme activity was expressed as micromoles of succinate oxidized per minute per milligram protein using a molar extinction coefficient of 1000 M^−1^ cm^−1^.

#### Cytochrome *c* reductase (complex III)

Cytochrome *c* reductase activity was measured as described by Waseem and Parvez (2016). The results were expressed as micromoles of formazan formed per minute per milligram protein with a molar excitation coefficient of 51,000 M^−1^ cm^−1^.

#### F1-F0 synthase activity (complex V)

ATP synthase is also referred to as mitochondrial complex V. Its activity was assayed as hydrolysis of ATP into ADP plus inorganic phosphate (P_i_) as described by Waseem and Parvez (2016). The enzyme activity was expressed as microgram of P_i_ liberated per minute per milligram protein.

### Assessment of oxidative stress damage

Mitochondrial LPO was assayed according to the procedure of Chaudhary and Parvez (2012) with certain modifications. The results were expressed as nanomoles of thiobarbituric acid-reactive substances (TBARS) formed per hour per gram tissue using a molar extinction coefficient of 1.56×10^−5^ M^−1^ cm^−1^. GSH concentration in brain mitochondria was measured according to the method of Tabassum et al. (2007). The GSH content was calculated as nanomoles of GSH reduced per gram tissue.

### Mitochondrial respiration measurement

Mitochondrial oxygen consumption was measured by using a Clark-type oxygen electrode (Hansatech Instrument) (Waseem and Parvez, 2016) at 37°C pH 7.4 in a KCl medium containing 0.1 mM EDTA, MgCl_2_, sucrose and KH_2_PO_4_. Rats were sacrificed 24 h after tMCAO and frontal cortex was isolated for mitochondrial preparations. Mitochondrial respiratory energy coupling was evaluated by determining respiratory control ratio (RCR) calculated as the rate of ADP-induced state 3 respiration to the state 4 rate without ADP. The rate of mitochondrial respiration was measured as nanomoles of oxygen (O_2_)/min/mg of protein.

### Mitochondrial swelling

Mitochondrial swelling caused by the influx of solutes through open permeability transition pores results in an increase in light transmission (i.e. a reduced turbidity). This turbidity change offers a convenient and frequently used assay of the mitochondrial permeability transition by measurement of absorbance in mitochondrial suspensions. Mitochondrial permeability was assayed by Ca^2+^-induced mitochondrial swelling and was assayed by spectrophotometry (Li et al., 2012). The mitochondrial pellet was re-suspended in ice-cold BSA-free and EDTA-free sucrose buffer after the last step of washing (300 mmol sucrose and 10 mmol/l Tris-Base, pH 7.4). The aliquot of 100 µg of mitochondria was added to 1 ml of BSA-free and EDTA-free buffer and 400 µm Ca^2+^ was added after 5 min and reading was taken for 5 min at 540 nm.

### Flow cytometric analysis of MMP and mitochondrial ROS

Flow cytometry analysis was performed using a FACSCalibur equipped with a 488 nm argon laser and a 635 nm red diode laser according to Li et al. (2012). Data from the experiments were analyzed using the CellQuest software (BD Bioscience). To exclude debris, samples were gated based on light scattering properties in the side scattering (SSC) and forward scattering (FSC) modes, and 10,000 events per sample within the R1 gate were collected. The mitochondrial sample was suspended in analysis buffer containing 250 mmol/l sucrose, 20 mmol/l MOPS, 10 mmol/l Tris-base, 100 µmol/l Pi(K), and 0.5 mmol/l Mg^2+^ and 5 mmol/l succinate at pH 7.4. The mitochondria were then stained with TMRE (100 nmol/l, excitation at 488 nm and emission at 590 nm) and H2DCFDA (2, 7 dichloro dihydro fluorescien diacetate) (10 mmol/l, excitation at 488 nm and emission at 525 nm), which were used to measure the MMP and the production of ROS, respectively.

### Neurotransmitter detection using HPLC

Estimation of 5-HT and dopamine in the brain frontal cortex was done using high-performance liquid chromatography (HPLC)-electrochemical detection (ECD) by the method of Chaudhary and Parvez (2012). Chromatographic analyses were performed at room temperature. Data were acquired and processed in the Empower Pro Operating System. 5-HT peaks were identified by comparing their retention time in the sample and its concentration was estimated according to the area under the curve using the straight line equation *y*=*mx*+*c*. Dopamine concentration was represented as ng/mg protein.

### Neuronal insult markers

MAO was measured according to the method reported by Vishnoi et al. (2015). The enzyme activity was assayed as nanomoles of benzyl amine hydrochloride (BAHC) hydrolyzed per minute per milligram protein using a molar extinction coefficient of 7.6925 M^−1^ cm^−1^. AchE was estimated according to the method of Vishnoi et al. (2015). The enzyme activity was measured as nmoles of acetyl thiocholine (ATC) hydrolyzed/min/mg protein using a molar extinction coefficient of 1.36×10^4^ M^−1^ cm^−1^. Na^+^ K^+^-ATPase activity was measured according to the method developed by Chaudhary and Parvez (2012). The activity was measured as µg of P_i_ liberated/min/mg protein.

### Histology

After performing neurobehavioral tests, animals were deeply anesthetized intraperitoneally with chloral hydrate (400 mg/kg b.w.) at 24 h after ischemia-reperfusion and were transcardially perfused through the ascending aorta with 0.9% saline according to the method of Ashafaq et al. (2012a). This was followed by ice-cold buffered 4% paraformaldehyde and then the brain was removed and put in paraformaldehyde for 48 h. Brain removal was followed by dehydration with ethanol. It was then embedded in paraffin then coronal sections (40 µm thick) were taken for hematoxylin and eosin staining. Histopathological scoring was done with the aid of a pathologist in our animal house. Scoring was done on the basis of morphological changes in the frontal cortex. According to the morphological changes, sections were given scores as follows: 0 (no change), 1 (minor changes), 2 (vacuolated spaces), 3 (pyknotic nuclei), 4 (vacuolated spaces and pyknotic nuclei), 5 (heavy neuronal loss).

### Cytochrome *c*

Immunohistochemical analysis was performed according to the method of Vishnoi et al. (2015), with some modifications. The brain was removed and fixed in 4% paraformaldehyde. Brain sections were cut from paraffin-embedded, paraformaldehyde-fixed brain tissue and mounted on poly-L-lysine-coated microscopic slides. Sections were deparaffinized three times (5 min) in xylene followed by dehydration in graded ethanol and finally rehydrated in running tap water. For antigen retrieval, sections were boiled in 10 mM citrate buffer (pH 6.0) for 10-15 min. Sections were incubated with hydrogen peroxide for 15 min to minimize non-specific staining and then rinsed three times (5 min each) with PBST (0.05% Tween-20). Blocking solution was applied for 10 min, then sections were incubated with the anti-cytochrome *c* antibody mouse monoclonal (dilution 1:200, Calbiochem^®^, cat. #QIA87) overnight at 4°C in a humid chamber. The anti-cytochrome *c* antibody is a mouse monoclonal (isotype IgG2b) that reacts with human, mouse and rat cytochrome *c*. The next day, the slides were washed with PBST three times and were incubated with secondary antibody goat anti-mouse IgG for 2 h. The peroxide complex was visualized with 3,3-diaminobenzidine (DAB). Next, the slides were counterstained with hematoxylin and dried. Finally, the sections were mounted with dibutylphthalate polystyrene xylene and covered with coverslips. The slides were then ready to be observed under the microscope. The intensity of the cytosolic immunostaining protocol was used for quantitative evaluation of immunostaining. Measurements were carried out using an inverted light microscope using objectives with 40× magnifications. Quantification of immunohistochemistry slides was performed by ImageJ 1.49 V (Wayne Rasband National Institute of Health, USA).

### Determination of protein

Protein contents in different fractions (supernatant, homogenate and mitochondria) of the brain frontal cortex were assayed by the Bradford method using BSA as a standard.

### Statistical analysis

All data are shown as mean±standard error of mean (s.e.m.). All data were analyzed by using analysis of variance (ANOVA) followed by Tukey's test. All data were analyzed by using GraphPad Prism 5 software (GraphPad Software Inc., San Diego, CA, USA). Values of *P*<0.05 were considered significant.
